# Effect of plate working length on plate stiffness and cyclic fatigue life in a cadaveric femoral fracture gap model stabilized with a 12-hole 2.4 mm locking compression plate

**DOI:** 10.1186/1746-6148-9-125

**Published:** 2013-06-24

**Authors:** Peini Chao, Bryan P Conrad, Daniel D Lewis, MaryBeth Horodyski, Antonio Pozzi

**Affiliations:** 1 Comparative Orthopaedics Biomechanical Laboratory, University of Florida, Gainesville, FL, USA; 2 Department of Small Animal Clinical Sciences, College of Veterinary Medicine, University of Florida, Gainesville, FL, USA; 3 Department of Orthopaedics and Rehabilitation, College of Medicine, University of Florida, Gainesville, FL, USA

**Keywords:** Locking compression plate, Femur, Fatigue, Stiffness, Gap motion, Dog

## Abstract

**Background:**

There are several factors that can affect the fatigue life of a bone plate, including the mechanical properties of the plate and the complexity of the fracture. The position of the screws can influence construct stiffness, plate strain and cyclic fatigue of the implants. Studies have not investigated these variables in implants utilized for long bone fracture fixation in dogs and cats. The purpose of the present study was to evaluate the effect of plate working length on construct stiffness, gap motion and resistance to cyclic fatigue of dog femora with a simulated fracture gap stabilized using a 12-hole 2.4 mm locking compression plates (LCP). Femora were plated with 12-hole 2.4 mm LCP using 2 screws per fracture segment (long working length group) or with 12-hole 2.4 mm LCP using 5 screws per fracture segment (a short working length group).

**Results:**

Construct stiffness did not differ significantly between stabilization techniques. Implant failure did not occur in any of the plated femora during cycling. Mean ± SD yield load at failure in the short plate working length group was significantly higher than in the long plate working length group.

**Conclusion:**

In a femoral fracture gap model stabilized with a 2.4 mm LCP applied in contact with the bone, plate working length had no effect on stiffness, gap motion and resistance to fatigue. The short plate working length constructs failed at higher loads; however, yield loads for both the short and long plate working length constructs were within physiologic range.

## Background

There are several factors that can affect the fatigue life of the plate. In addition to the mechanical properties of the isolated plate, the location, type and complexity of the fracture influence the load acting on the plate [1-6]. Bridging plates are often utilized in comminuted fractures to span a large unreconstructed segment of the fractured bone, resulting in a long segment of plate unsupported by screws [7]. The distance between the proximal and distal screw in closest proximity to the fracture is defined as the “working length” of the plate. Plate working length has been shown to influence construct stiffness, plate strain and cyclic fatigue properties of the plate [5,8-11]. The lack of load sharing between the stabilized bone and the implants increases the risk of cyclic fatigue and early failure of the implant [9,12]. A mechanical study evaluating the mechanical endurance of human femora stabilized with 14-hole broad 4.5 mm LCPs found that constructs with load sharing resisted 20 times more cycles than the constructs with an 8 mm segmental diaphyseal gap [13].

Controversy still exists regarding the effect of plate working length on stiffness and resistance to fatigue failure [5,12,14]. Stoffel et al. reported that increasing the plate working length by omitting one screw placed adjacent to the fracture in each of the major fracture segments made locking plating constructs nearly twice as flexible when loaded in compression and torsion [12]. In contrast, Field et al. reported that omitting two screws proximal and distal to the fracture had no significant effect on either bending or torsional stiffness of conventional plate constructs in a comparable bridge plate configuration [14]. Studies evaluating the effect of working length on the cyclic fatigue properties of plated constructs have yielded inconsistent results [5,9,12]. Stoffel et al. found that the constructs with shorter plate working length were more resistant to cyclic loading [12]. Maxwell et al. reported similar results when screw placement was evaluated in 3.5 mm dynamic compression plates and limited contact dynamic compression plates applied in a fracture gap model [11]. Recent studies comparing the cyclic fatigue properties of plates applied with a short and long working length found that the constructs with a longer working length withstood more cycles before failure, although the differences between stabilization techniques were not significant [5,9].

The purpose of the present study was to evaluate the effect of plate working length on construct stiffness, gap motion and resistance to cyclic fatigue of dog femora with a simulated fracture gap stabilized using a 12-hole 2.4 mm LCP. Our first hypothesis was that constructs with a longer plate working length would be more flexible and have greater gap motion than constructs with a shorter plate working length. The second hypothesis was that constructs with a longer plate working length would withstand more cycles before succumbing to fatigue failure than constructs with a shorter plate working length.

## Methods

### Specimen preparation

This study was approved by the University of Florida Institutional Animal Care and Use Committee (#201105539). Ten pairs of femora were collected from adult dogs (mean body weight ± SD: 17.9 ± 1.9 kg) that were euthanized for reasons unrelated to the study. Once all of the soft tissues were removed from the femurs, the bones were radiographed in order to exclude specimens with osseous abnormality. The femora were then wrapped in saline-soaked towels and stored at -20°C until testing, at which time the bones were thawed to room temperature. Randomized within each pair of femurs, one femur was assigned to the short plate working length stabilization group. The contralateral femur was assigned to the long plate working length stabilization group.

**Figure 1:**
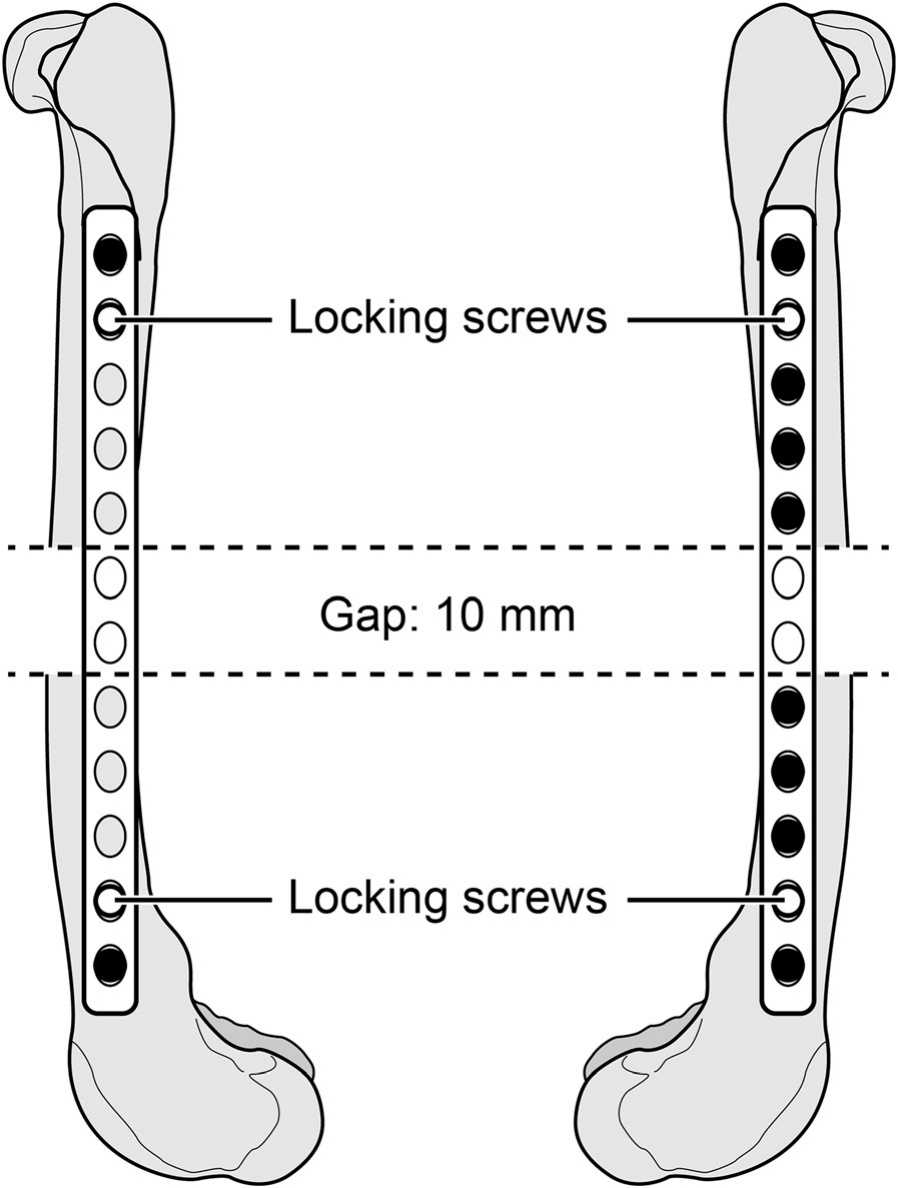
**Testing constructs of long and short plate working length stabilization techniques.** Long plate working length stabilization technique (left) and short plate working length stabilization technique (right). Each plate had a 2.4 mm locking screw (○) placed in the second hole from each end of the plate. The other screws were 2.4 mm cortical screws (●).

A 12-hole 2.4 mm LCP (Synthes USA, Paoli, PA) was contoured and clamped temporarily to the lateral surface of each femur. Two lines, 10 mm apart were marked on the mid-diaphyseal region of the bone adjacent to the central 2 holes of the plate. The plate was removed, and an oscillating saw was used to initiate two mid-diaphyseal partial osteotomies in the lateral cortex of the femur at the marked locations. In the femora assigned to the long plate working length group, the plate was affixed in contact with the femur with one 2.4 mm cortical and one 2.4 mm locking screw placed at the ends of the plates, leaving eight empty holes in the middle of the plate (Figure 1). The femora assigned to the short plate working length group were stabilized in direct contact with the femur with four 2.4 mm cortical and one 2.4 mm locking screws in each femoral segment, leaving two empty holes in the center of the plate (Figure 1). All screws had bicortical purchase and were placed in the same order. Cortical screws at each end of the plate were placed first, followed by the locking screw in the second hole and then the remaining cortical screws. All screws were placed and hand-tightened by an experienced board-certified surgeon (AP) in accordance with standard AO-ASIF techniques [15]. Prior to testing, each locking and cortical screw were tightened at a torque of 0.8 Nm or 0.4 Nm, respectively. After applying all screws, osteotomies were completed from the medial cortex of the femur resulting in a 10 mm-long mid-diaphyseal segmental ostectomy.

The distal ends of femora were potted using epoxy resin (Bondo Corp, Atlanta, GA). A goniometer was used to ensure that the specimens were potted perpendicular to the testing mold. Two points located on the cranial and caudal surface of each femur segment adjacent to the fracture gap were first marked then scribed using a 1.5 mm drill bit, so these points could be accurately identified with the tip of the articulating arm of a three-dimensional positioning device (Microscribe 3DX digitizing arm, Immersion Corp., San Jose, CA).

### Mechanical testing

All testing was performed using a servohydraulic mechanical testing machine (MTS 858 Mini Bionix II, MTS Systems Corp, Eden Prairie MN). During testing, each specimen was positioned vertically with the epoxy resin block secured to the machine using a specially designed fixture. A polyoxymethylene cup simulating the shape of the acetabulum was secured to the MTS and used to load the femora. In order to measure gap displacement of the bone segments, three points on each bone fragment were digitized using the Microscribe unit while the limb was preloaded to 10 N.

A cyclic axial compressive load from 0 N to 100% body weight (mean ± SD: 175.5 ± 18.6 N) was applied at 2 Hz, while displacement data was recorded at 100 Hz throughout testing. Testing was paused at 1000, 2000, 6000, 10000, 20000, 40000, 60000, 120000, 180000 cycles to obtain gap displacement measurements as each specimen was loaded. If fatigue failure did not occur after 180,000 cycles, the specimen was loaded in axial compression at a rate of 1 mm/seconds until failure was achieved. Displacement data was recorded at 100 Hz while specimens were loaded to failure. Failure was defined as breakage or plastic deformation of the implants or any visually detected loosening of screws in the plate or bone. Video was recorded during testing to identify the mode of failure for each construct.

### Data and statistical analysis

Displacement values were recorded at 1000, 2000, 5000, 10000, 20000, 50000, 100000, 120000, and 18000 cycles. A load–displacement curve was calculated by plotting the load versus displacement data for each specimen for each time point during cyclic testing in a scatter graph using Microsoft Office Excel (Microsoft Corporation, Redmond, Washington). A best fit trend line, straight line slope, and corresponding R^2^ value were determined for each load–displacement curve, using a sum of least squares method for the linear elastic region of the load–displacement curve using the same software. The slope of this line defined the stiffness value and was reported in units of N/mm.

Relative gap displacement of two bone segments in the sagittal and frontal plane was analyzed using classical principles of rigid body mechanics [16]. Gap displacement of each construct was also calculated to compare whether there was any significant difference between the short and long plate working length stabilization techniques. Calculations were performed using a custom-written computer program (MATLAB®, MathWorks Corporation, Natick, Massachusetts).

A separate load-deformation curve was determined similarly from the initial linear part of load to failure test for each construct. The elastic limit or yield load was defined as the loading value at which the linear phase of the curve terminated [17]. Mean values and standard deviations of stiffness, gap displacement and yield load were determined for both the short and long plate working length stabilization techniques.

Stiffness values, cranial/caudal translation and medial/lateral translation of the two bone segments and gap displacement measured during cyclic testing were compared between the short and long plate working length groups using a 2 × 9 (technique x cycle) within subjects repeated measures ANOVA. When appropriate a post hoc Bonferroni procedure was used to adjust for multiple pairwise comparisons (SPSS Statistics 17.0, SPSS Inc., Chicago, IL). The yield load determined during load to failure testing was statistically compared between the short and long plate working length stabilization techniques using a paired *t*-test. For all statistical analyses, significance was set at *P* < 0.05.

## Results

**Figure 2:**
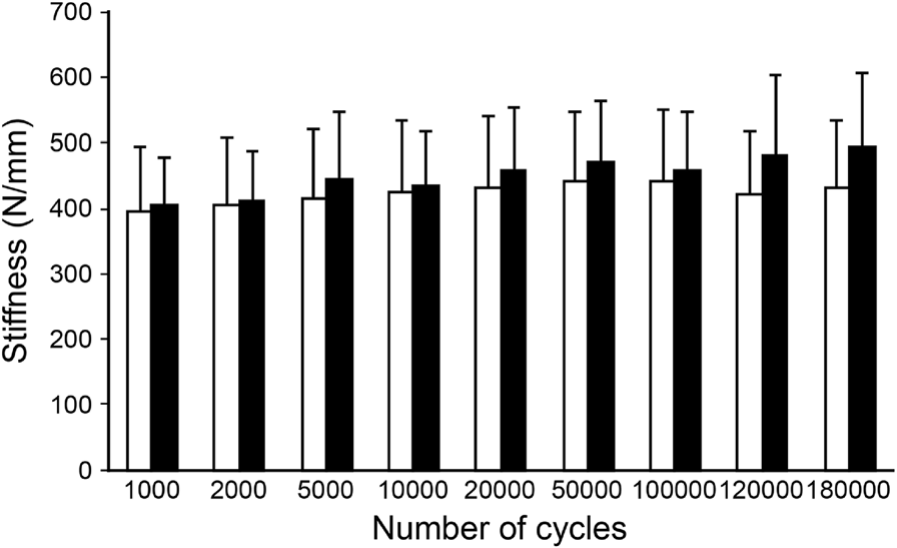
**Testing results between long and short plate working length stabilization techniques.** Comparison of mean ± SD values of construct axial stiffness (N/mm) loaded to body weight between the long plate working length stabilization technique (□) and the short plate working length stabilization technique (■). Measurements recorded at 1000, 2000, 5000, 10000, 20000, 50000, 100000, 120000, and 180000 cycles. No significant difference was found between the two stabilization techniques at any of the evaluated cycles.

When the constructs were cyclically loaded to the dog’s body weight, there was no statistically significant difference in stiffness between the short and long plate working length stabilization techniques (*P* = 0.435). There was a trend for the stiffness to increase during cycling in both the short and long working length plate groups until 50,000 cycles. After reaching 50,000 cycles, the mean stiffness of the long working length plate group decreased while the mean stiffness of the short working length plate group continued to increase, although the difference was not significant between stabilization techniques (Figure 2). All constructs completed 180,000 cycles of loading without plate or screw breakage, screw loosening or visible plastic deformation of the implants observed in any of the constructs.

There were no significant differences in the relative displacement of the bone segments between stabilization techniques in the sagittal (*P* = 0.448) or frontal plane (*P* =0.504). Gap displacement also did not differ significantly within the designated cycles and between stabilization techniques (*P* =0.116). The ends of the two stabilized bone segments did not come into contact in any of the constructs during cyclic compressive loading.

**Figure 3:**
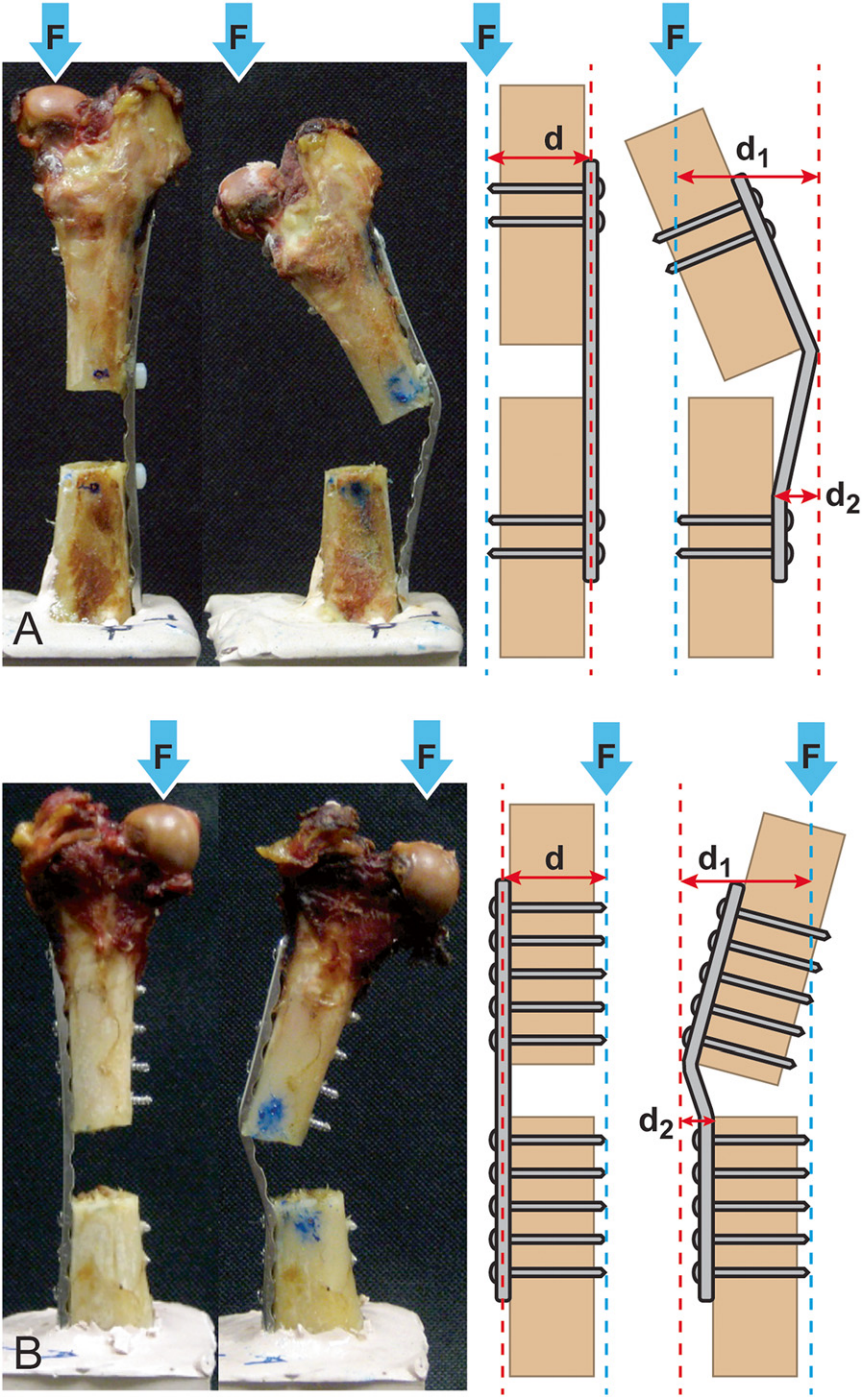
**Photographs and free body diagrams of a long (A) and short (B) plate working length construct before and after load to failure test.** Photographs of a long **(A)** and short **(B)** plate working length construct before (left) and after (right) load to failure testing. The adjacent free body diagrams represent how bending moments were created. Bending moments (Force (F) x Distance (d)) were calculated based on measured scale distances obtained from photographs and the yield load of each construct. The off axis shift of the femoral head observed in the figure may be due to elastic displacement after unloading the specimen, as the pictures are taken without load.

During load to failure testing, constructs in the short plate working length group had a significantly (*P* =0.016) higher yield load (mean ± SD: 654.5 ± 149.0 N) than constructs in the long plate working length group (mean ± SD: 452.9 ± 63.4 N). The mean ± SD stiffness of the short (223.8 ± 46.7 N/mm) and long (219.4 ± 80.3) plate working length groups during load to failure testing was not significantly different. Failure patterns for the short and long plate working length constructs were distinctively different (Figure 3): the proximal femoral segment bent medially during axial loading in both the short and long plate working length constructs. The lateral surface of the proximal femoral segment acted as a fulcrum against the plate and there was no separation of the plate from the lateral cortex of the femur in both groups. The plate elevated from the lateral cortex of the distal femoral segment in the long plate working length group. The plate remained apposed to the lateral cortex of the femur in the short plate working length group.

Review of video obtained during the load to failure testing revealed that eccentric axial compressive load applied to the femoral head leads to the formation of a bending moment, with the center of rotation located at the plate-bone interface. Bending moment was defined as the product of applied force (F) amount and the perpendicular distance (d) from the applied force to the axis of rotation (Figure 3). The bending moment increased during the load to failure test as the plate deflected away from the lateral cortex of the femur. This effect was more pronounced in the long plate working length constructs.

## Discussion

Increasing working length by placing a limited number of screws at each end of the plate has been recommended as a strategy to decrease construct stiffness and therefore allow more motion at the fracture gap [1,7,12,18,19]; however, previous mechanical studies have yielded conflicting results [8,9,11,12]. Based on our results, we cannot support our hypotheses that constructs with fewer screws and a longer plate working length would be more flexible, have greater gap motion and be more resistant to cyclic fatigue failure than constructs with more screws and a short plate working length. We suspect that the inconsistencies in the results amongst reported studies might be attributed to the manner in which the plate is applied to the bone. In constructs that utilize non-locking plates [11,14], the contact between the plate and the bone segments causes the bending moment to concentrate between the ends of the bone segments regardless the positioning of the screws. Therefore, the functional plate working length was not equal to the distance between the screws placed closest to the fracture gap, but rather to the unsupported area of the plate, which corresponded with the length of the fracture gap in this study. In contrast, the physical offset of a locking plate which is applied without the bone and plate in intimate contact enables a locking plate to bend along the entire segment of the plate between the two most centrally positioned screws [12]. If our suppositions are confirmed, the concept of short and long plate working length as the distance between the screws closest to the fracture should only be applied to true functional locking plate constructs and should not be applied to conventional non-locking plate constructs.

The results of our cyclic fatigue testing are not consistent with previous studies which found differences between constructs with short and long plate working lengths [11,12]. These contrasting results might be ascribed to differences in the fracture models employed and methods of loading. In our study the applied load was based on the dogs’ body weight in an attempt to replicate clinical situations. We used a similar approach to previous mechanical tests performed in canine femora. Goh et al. tested plated femora using 20%, 40%, and 60% of body weight to simulate progressively increased weight bearing in the early postoperative period, using values based on kinetic studies in dogs [20,21]. As 40% to 50% of body weight is achieved in the hind limbs of clinically normal dogs during walking, we would expect that 100% of body weight would be a reasonable load to challenge the implant resistance to cyclic fatigue [21]. In other studies the upper load threshold was set to induce implant failure within a predetermined number of cycles [5] or was established using displacement control based on plate strain [9]. Establishing significance may have been masked in our study because we did not apply high enough loads as none of our constructs failed during cyclic testing. The magnitude of the load applied in diaphyseal fracture models is more complex than simply applying a load equivalent to mean body weight. In human patients which have been instrumented with distal femoral diaphyseal prosthesis with telemetric strain gauges, load values as high as three times body weight and bending forces up to ten times body weight have been recorded [22]. Other loading protocols such as progressive loading [23] could have been used to shorten the test protocol and may have yielded significant differences between stabilization techniques.

Both the short and long plate working length stabilization constructs sustained 180,000 cycles with the equivalent of full body weight loaded in axial compression without failure, which approximates 2 to 4 months of weight-bearing during normal ambulation [24]. This finding suggests that both constructs would likely provide acceptable clinical stability as most fractures are expected to obtain union within 12 weeks of stabilization [25,26]. The yield load for both stabilization techniques ranged from 453 to 655 N, which corresponds to 2 to 4 times the hind limbs peak vertical force measured from a normal 20 kg dog ambulating at a trot [20,27]. Although kinetic values measured with gait analysis are only an approximation of the forces tolerated by implants during loading, our results suggest that it is unlikely that either of our constructs would fail catastrophically in the early postoperative convalescent period. However, when interpreting our results it should be considered that we did not apply torsional loads, which may have contributed to earlier failure, especially in the long plate working length stabilization group.

Cadaveric studies have numerous limitations. When performing cyclic testing designated to resemble a clinical environment after fracture fixation, the loading plane should be considered [28]. We selected an offset axial loading to simulate loading of a plated femoral fracture. Our testing methodology had the limitation of being isolated to a single plane, without considering more complex forces such as a combination of bending and torsional forces. In the diaphyseal region of the femur, however, axial and bending forces predominate and these forces were replicated in our testing protocol [22]. Additionally, constraining the distal femur, as previously described [20], may have influenced the mode of failure. We, however, utilized uniform testing conditions allowing for valid comparisons between treatment groups. Another limitation was that we did not test a construct which utilized only locking screws. This omission limits our ability to make conclusions regarding the effect of plate working length of a locking plate employed in its intended application [9,12]. A hybrid construct in which both locking and non-locking screws were used was selected because these constructs are commonly used in dogs [29]. Furthermore, previous studies have shown that placement of a single locking screw in each of the major fracture segments increases a construct’s axial and torsional stability [29-32]. Another limitation that should be considered is that constructs had differences in the number of screws, in addition to differences in plate working length. Results of previous mechanical studies suggest that the number of screws may be a less important variable than the position of the screws and plate length [4,12]. Our intent was to evaluate two constructs that represented contrasting approaches to the stabilization of long bone fractures in dogs [33,34].

## Conclusions

We were unable to establish a significant effect of plate working length on the stiffness and resistance to cyclic fatigue in a fracture gap model plated with 2.4 mm LCP applied in direct cortical contact with the femur. Our results question the purported advantage of long plate working length in decreasing stiffness of the bone-plate construct and protecting the portion of unsupported plate at the fracture gap [18,19,35]. Neither of the constructs tested in our study failed during cyclic load. Therefore both short and long plate working length constructs would appear to be acceptable for clinical applications. Placing five bicortical screws in each of the major fracture segments may be unnecessary. Other studies have reported that fewer, more widely spaced screws increased the bending strength of screw–plate fixation more effectively than increasing the number of screws [1,3,19]. Guidelines regarding the optimal number of screws will, however, need to be derived from future biomechanical and clinical studies.

## Competing interests

None of the authors believe their interpretation or presentation of the data was influenced by any financial competing interests. Dr. Antonio Pozzi has been paid in sponsored Continuing Education seminars with SynthesVet and received funding from SynthesVet for previous projects. This study was supported by University of Florida faculty start-up development funds.

## Authors’ contributions

PC, BPC, DDL and AP conceived and designed the study; PC, BPC and AP collected the data; MBH performed the statistical analysis; AP, BPC and DD interpreted the results; PC and AP drafted the manuscript; all authors read, contributed to and approved the final manuscript.

## References

[B1] Gautier E Sommer C Guidelines for the clinical application of the LCP Injury 2003 34 Supplement 2 63 76 10.1016/j.injury.2003.09.026 14580987

[B2] Miller DL Goswami T A review of locking compression plate biomechanics and their advantages as internal fixators in fracture healing Clin Biomech 2007 22 10 1049 1062 10.1016/j.clinbiomech.2007.08.004 17904257

[B3] Tornkvist H Hearn TC Schatzker J The strength of plate fixation in relation to the number and spacing of bone screws J Orthop Trauma 1996 10 3 204 208 8667113 10.1097/00005131-199604000-00009

[B4] Sanders R Haidukewych GJ Milne T Dennis J Latta LL Minimal versus maximal plate fixation techniques of the ulna: the biomechanical effect of number of screws and plate length J Orthop Trauma 2002 16 3 166 171 11880779 10.1097/00005131-200203000-00005

[B5] Hoffmeier KL Hofmann GO Mückley T Choosing a proper working length can improve the lifespan of locked plates: a biomechanical study Clin Biomech 2011 26 4 405 409 10.1016/j.clinbiomech.2010.11.020 21185629

[B6] Hammel SP Elizabeth Pluhar G Novo RE Bourgeault CA Wallace LJ Fatigue analysis of plates used for fracture stabilization in small dogs and cats Vet Surg 2006 35 6 573 578 16911158 10.1111/j.1532-950X.2006.00191.x

[B7] Kubiak EN Fulkerson E Strauss E Egol KA The evolution of locked plates J Bone Joint Surg Am 2006 88 suppl_4 189 200 17142448 10.2106/JBJS.F.00703

[B8] Ellis T Bourgeault CA Kyle RF Screw position affects dynamic compression plate strain in an in vitro fracture model J Orthop Trauma 2001 15 5 333 337 11433137 10.1097/00005131-200106000-00005

[B9] Kanchanomai C Muanjan P Phiphobmongkol V Stiffness and endurance of a locking compression plate fixed on fractured femur J Appl Biomech 2010 26 10 16 20147753 10.1123/jab.26.1.10

[B10] Korvick DL Monville JD Pijanowski GJ Phillips JW The effects of screw removal on bone strain in an idealized plated bone model Vet Surg 1988 17 3 111 116 3238883 10.1111/j.1532-950x.1988.tb00288.x

[B11] Maxwell M Horstman CL Crawford RL Vaughn T Elder S The effects of screw placement on plate strain in 3.5 mm dynamic compression plates and limited-contact dynamic compression plates Vet Comp Orthop Traumatol 2009 22 125 131 19290393 10.3415/vcot-08-02-0023

[B12] Stoffel K Dieter U Stachowiak G Gächter A Kuster MS Biomechanical testing of the LCP - how can stability in locked internal fixators be controlled? Injury 2003 34 Supplement 2 11 19 10.1016/j.injury.2003.09.021 14580982

[B13] Kanchanomai C Phiphobmongkol V Muanjan P Fatigue failure of an orthopedic implant - a locking compression plate Eng Fail Anal 2008 15 5 521 530

[B14] Field JR Tornkvist H Hearn TC Sumner-Smith G Woodside TD The influence of screw omission on construction stiffness and bone surface strain in the application of bone plates to cadaveric bone Injury 1999 30 9 591 598 10707226 10.1016/s0020-1383(99)00158-8

[B15] Koch D Johnson AL, Houlton JEF, Vannini R Screws and plates AO principles of fracture management in the dog and cat 2005 New York: Thieme 27 51

[B16] Crane CD III Duffy J Manipulator kinematics Kinematic analysis of robot manipulators 2008 New York: Cambridge University Press 33 36

[B17] Hvid I Jensen J Cancellous bone strength at the proximal human tibia Engineering in Medicine 1984 13 1 21 25 6538519 10.1243/emed_jour_1984_013_007_02

[B18] Lill H Hepp P Korner J Kassi JP Verheyden AP Josten C Duda GN Proximal humeral fractures: how stiff should an implant be? Archives of Orthopaedic and Trauma Surgery 2003 123 2 74 81 12721684 10.1007/s00402-002-0465-9

[B19] Wagner M General principles for the clinical use of the LCP Injury 2003 34 31 42 10.1016/j.injury.2003.09.023 14580984

[B20] Goh CS Santoni BG Puttlitz CM Palmer RH Comparison of the mechanical behaviors of semicontoured, locking plate-rod fixation and anatomically contoured, conventional plate-rod fixation applied to experimentally induced gap fractures in canine femora Am J Vet Res 2009 70 1 23 29 19119945 10.2460/ajvr.70.1.23

[B21] DeCamp CE Kinetic and kinematic gait analysis and the assessment of lameness in the dog Vet Clin North Am Small Anim Pract 1997 27 4 825 840 9243783 10.1016/s0195-5616(97)50082-9

[B22] Taylor SJG Walker PS Forces and moments telemetered from two distal femoral replacements during various activities J Biomech 2001 34 7 839 848 11410168 10.1016/s0021-9290(01)00042-2

[B23] Bottlang M Doornink J Fitzpatrick DC Madey SM Far cortical locking can reduce stiffness of locked plating constructs while retaining construct strength J Bone Joint Surg Am 2009 91 8 1985 1994 19651958 10.2106/JBJS.H.01038 PMC2714811

[B24] Aper RL Litsky AS Roe SC Johnson KA Effect of bone diameter and eccentric loading on fatigue life of cortical screws used with interlocking nails Am J Vet Res 2003 64 5 569 573 12755296 10.2460/ajvr.2003.64.569

[B25] Claes L Heitemeyer U Krischak G Braun H Hierholzer G Fixation technique influences osteogenesis of comminuted fractures Clin Orthop Relat Res 1999 365 221 229 10627706 10.1097/00003086-199908000-00027

[B26] Baumgaertel F Buhl M Rahn BA Fracture healing in biological plate osteosynthesis Injury 1998 29 Supplement 3 3 6 10.1016/s0020-1383(98)95002-1 10341891

[B27] Bertram JEA Lee DV Case HN Todhunter RJ Comparison of the trotting gaits of Labrador Retrievers and Greyhounds Am J Vet Res 2000 61 7 832 838 10895909 10.2460/ajvr.2000.61.832

[B28] Gardner MJ Silva MJ Krieg JC Biomechanical testing of fracture fixation constructs: variability, validity, and clinical applicability J Am Acad Orthop Surg 2012 20 2 86 93 22302446 10.5435/JAAOS-20-02-086

[B29] Gordon S Moens NMM Runciman RJ Monteith GM The effect of the combination of locking screws and non-locking screws on the torsional properties of a locking-plate construct Vet Comp Orthop Traumatol 2010 23 7 13 19997676 10.3415/VCOT-09-05-0055

[B30] Gardner MJ Griffith MH Demetrakopoulos D Brophy RH Grose A Helfet DL Lorich DG Hybrid locked plating of osteoporotic fractures of the humerus J Bone Joint Surg Br 2006 88 1962 1967 10.2106/JBJS.E.00893 16951112

[B31] Goswami T Patel V Dalstrom DJ Prayson MJ Mechanical evaluation of fourth-generation composite femur hybrid locking plate constructs Journal of Materials Science: Materials in Medicine 2011 22 9 2139 2146 21769628 10.1007/s10856-011-4388-2

[B32] Freeman AL Tornetta P III Schmidt A Bechtold J Ricci W Fleming M How much do locked screws add to the fixation of “hybrid” plate constructs in osteoporotic bone? J Orthop Trauma 2010 24 3 163 169 20182252 10.1097/BOT.0b013e3181d35c29

[B33] Johnson AL Smith CW Schaeffer DJ Fragment reconstruction and bone plate fixation versus bridging plate fixation for treating highly comminuted femoral fractures in dogs: 35 cases (1987-1997) J Am Vet Med Assoc 1998 213 8 1157 1161 9787384

[B34] Cabassu JP Elastic plate osteosynthesis of femoral shaft fractures in young dogs Vet Comp Orthop Traumatol 2001 14 40 45

[B35] Smith WR Ziran BH Anglen JO Stahel PF Locking plates: tips and tricks J Bone Joint Surg Am 2007 89 10 2298 2307 17966158 10.2106/00004623-200710000-00028

